# Is the Association Between Early Childhood Screen Media Use and Effortful Control Bidirectional? A Prospective Study During the COVID-19 Pandemic

**DOI:** 10.3389/fpsyg.2022.918834

**Published:** 2022-06-27

**Authors:** Caroline Fitzpatrick, Elizabeth Harvey, Emma Cristini, Angélique Laurent, Jean-Pascal Lemelin, Gabrielle Garon-Carrier

**Affiliations:** ^1^Faculty of Education, Université de Sherbrooke, Sherbrooke, QC, Canada; ^2^Department of Childhood Education, University of Johannesburg, Johannesburg, South Africa; ^3^Groupe de Recherche et d’Intervention sur les Adaptations Sociales de l’Enfance, Sherbrooke, QC, Canada; ^4^Department of Education, Université Sainte-Anne, Church Point, NS, Canada

**Keywords:** screen media, screen time, effortful control, early childhood, temperament, family adversity

## Abstract

Individual differences in effortful control, a component of temperament, reflecting the ability to use attention and other cognitive processes to self-regulate emotion and behavior, contribute to child academic adjustment, social competence, and wellbeing. Research has linked excessive screen time in early childhood to reduced self-regulation ability. Furthermore, research suggests that parents are more likely to use screens with children who have more challenging temperaments, such as low levels of effortful control. Since screen time by children between the ages of 0 and 18 has increased during the COVID-19 pandemic, it remains timely to investigate the developmental pattern of association between child screen media use and effortful control. We hypothesize that higher levels of screen media intake at age 3.5 will be associated with lower effortful control at age 4.5 and that lower effortful control at 3.5 will contribute to more screen media intake at age 4.5. This study draws on participants followed longitudinally over the span of 2-years for an investigation of Canadian preschoolers’ screen media use during the pandemic (*N* = 316, Wave 1). A follow-up with this sample was completed in 2021 (*N* = 265, Wave 2). Analyses using a cross-lagged panel model revealed stability in child screen time and effortful control between the ages of 3.5 and 4.5. Child screen time at age 3.5 significantly contributed to decreased effortful control scores at the age of 4.5, whereas effortful control at age 3.5 did not contribute to screen time at age 4.5. Our results partially confirmed our hypothesis and indicated that higher levels of screen time intake were detrimental to the development of effortful control. These results suggest that screen media use, an exceedingly frequent activity, may play an enduring role in development by shaping young children’s temperaments.

## Introduction

Child effortful control is a core component of temperament composed of attentional (e.g., attention focusing and shifting) and other cognitive (e.g., planning) skills that allow for the voluntary regulation of emotions and behaviors that may interfere with personal goals or environmental demands (Rothbart and Bates, 2006; Rothbart, 2011; Morris et al., 2013). Before children begin school, around the age of 3 and 4, effortful control contributes to their ability to benefit from informal learning situations and activities in the home and daycare setting (Liew, 2012; Merz et al., 2014). As children transition to school, effortful control is likely to help children succeed through a cascade of effects. For one, effortful control provides a strong basis for cognitive and social dimensions of school readiness (Potmesilova and Potmesil, 2021). Furthermore, better effortful control also contributes to child academic achievement indirectly through improved learning behaviors (Blair and Razza, 2007; Sánchez-Pérez et al., 2018). More specifically, child effortful control is associated with better classroom engagement and social competence, which each make contributions to academic achievement (Valiente et al., 2008; Sánchez-Pérez et al., 2018).

In addition, decades of research support the hypothesis that higher levels of child effortful control benefits mental health and wellbeing. Lower levels of effortful control are related to increased risk of behavior problems and psychopathology including aggression and antisocial behavior (Murray and Kochanska, 2002; Olson et al., 2005; Eisenberg et al., 2009, 2015; Gartstein et al., 2012; Wang et al., 2016; Diaz et al., 2017; Jonas and Kochanska, 2018; Smith and Day, 2018; Wichstrøm et al., 2018). To a lesser degree, lower levels of child effortful control have also been linked to increased risk of experiencing internalizing problems such as anxiety and depression (Santens et al., 2020). The ability to deploy attentional and cognitive resources to effectively control one’s behaviors and emotions during the preschool years may also foster benefits well beyond early childhood. In particular, research has found that higher levels of effortful control can provide children with long-term advantages including better health, financial successfulness, family stability, and lower risk of criminal conviction by adulthood, regardless of their IQ and parent’s social status (Moffitt et al., 2011).

### Effortful Control in the Context of Early Childhood Experience

Early childhood is a sensitive and foundational time for the strengthening of effortful control skills (Potmesilova and Potmesil, 2021). Between the ages of 3 and 4, child self-regulation skills evolve rapidly (Montroy et al., 2016). Individual differences in effortful control are in part driven by genetic differences and maturation (Diamond, 2002; Rothbart and Bates, 2006). Nevertheless, experiences and environments also play an essential and formative role in shaping children’s self-regulation skills (Rothbart, 2011; Tiberio et al., 2016). Longitudinal and experimental research indicate that sensitive caregiving and exchanges help children build these skills (Diamond et al., 2007; Blair and Raver, 2015; Landry et al., 2017; Nix et al., 2018; Warren and Barnett, 2020; Park et al., 2022). Raising children with low regulation skills can be especially challenging, even for the most sensitive, warm, and patient caregivers (Moffitt, 1993). Families that face higher levels of adversity in particular, are likely to experience challenges in providing the types of experiences that help build effortful control. As a result, disadvantaged children are more likely to develop lower levels of effortful control than their more advantaged peers (Lengua, 2012; Zalewski et al., 2012).

### Preschool Screen Time and Child Effortful Control

Screen time by young children has been linked to negative developmental outcomes (Pagani et al., 2013; Madigan et al., 2019), yet research has yet to examine its contribution to effortful control. From a prevention perspective, a focus on preschool children is advantageous because screen time habits adopted early on are likely to be carried forward later in life (Jones et al., 2013). Even though pediatric and health organizations recommend limiting screen time with preschool-aged children to 1 h a day, screen media use with preschool-aged children is increasingly common (Rideout, 2020). According to two Canadian studies conducted prior to the pandemic, only 46–58% of preschool-aged children respect the recommendation of <1 h/day of screen media (Tamana et al., 2019; Madigan et al., 2020).

There is evidence that non-adherence to pediatric screen time recommendations between the ages of 3 and 5 is associated with suboptimal development in the frontal-occipital fasciculus, a brain area involved in cognitive control (Hutton et al., 2020). Furthermore, *real world* longitudinal research supports these findings by indicating that children who accumulate too much time in front of screens may experience developmental delays across cognitive, social, and motor domains and are more at risk of arriving less well prepared to learn in kindergarten (Pagani et al., 2013; Madigan et al., 2019). Research has also linked early childhood screen time to reduced executive function ability in preschoolers (Nathanson et al., 2014; Ribner et al., 2017; Konok et al., 2021). More specifically, according to one cross-sectional study, the negative association between preschooler screen time and school readiness appears to be partially mediated by reduction in child executive functions (Ribner et al., 2017). Executive functions and effortful control both represent key mechanisms of self-regulation that share much overlap in their underlying neurological circuitry, developmental trajectories, function in modulating emotions and behavior, and measurement (Zhou et al., 2012).

These studies are consistent with displacement hypotheses. That is, too much media intake during a sensitive time for the development of self-regulation may create a time dept for other important experiences and activities. That is, media use may take time away from self-regulation building pursuits such as imaginary play, storytelling, or games that present motor challenges (Diamond and Lee, 2011). Given the importance of the preschool period for building the foundations of effortful control, devoting too much time to screen media use at the expense of other activities may be particularly costly at this age.

### Family Distress, the COVID-19 Pandemic, and Preschooler Screen Time

Research has found that families facing higher levels of adversity and who have less personal, social, and financial resources, are likely to expose children to more screen time (Hartshorne et al., 2021). Indeed, parents are likely to use more screens with children that are less-well regulated (Thompson et al., 2013; Coyne et al., 2021; Parrish et al., 2022). In addition, children with a low level of effortful control may have greater difficulty regulating emotional and physiological responses to media as well as disengaging from media (Clifford et al., 2020). Furthermore, screen-based activities generally require minimal effortful attention focusing from young children (Goodrich et al., 2009). For this reason, engaging young children in screen-based activities may place less strain on parents, particularly in the context of increased distress during the pandemic. Indeed, toddlers’ lower effortful control has been indirectly associated with greater screen use (Shin et al., 2021). Furthermore, research on 4–8-year-olds and older school-aged children has linked impulsivity and attention problems to more compulsive and problematic media use habits (Gentile et al., 2011; Paulus et al., 2018). The extent to which effortful control prospectively contributes to the development of screen time habits in young children during the pandemic remains to be examined.

In further support of the hypothesis that family distress contributes to child screen time, children in disadvantaged homes are more likely to spend time in front of screens, view developmentally inappropriate content, and view media without adult supervision (Wright et al., 2001; Asplund et al., 2015). Parent mental health may also contribute to parental practices surrounding child media use. For instance, parents who report being more stressed set less limits on their children’s screen time (Walton et al., 2014). To help inform effective child and family level interventions, it is therefore important to consider how family distress contributes to child screen time.

Research suggests that screen time has increased for children between the ages of 0 and 18 during the pandemic (Hartshorne et al., 2021). Parents are likely to have used more screen media during this time to keep young children busy or provide them with a respite from parenting responsibilities. Recent work from our group suggests that the majority (63%) of preschoolers were exposed to more than 2 h of screen time daily during the pandemic (Fitzpatrick et al., 2022). Furthermore, according to another recent study with Spanish children, accelerometer measured sedentary behavior and self-regulation problems have increased among preschoolers during the pandemic (Alonso-Martínez et al., 2021).

### The Current Study

The direction of the association between child screen time and effortful control, amidst increased screen media use by children during the pandemic remains unknown. Since early childhood represents a key developmental time for the strengthening of effortful control and the shaping of screen media habits, it is important to examine longitudinal associations between these variables. Previous research using cross-sectional and longitudinal designs has been unable to account for the direction of influence when examining associations between screen time and child outcomes. The present study attempts to address this limitations by simultaneously examining both directions of influence and by accounting for stability in screen time habits and effortful control. We predict that screen time at age 3.5 will be prospectively associated with lower levels of child effortful control by age 4.5. In addition, we hypothesize a bidirectional effect by which child effortful control at age 3.5 will predict screen time at age 4.5. Given that family distress is likely to have contributed to child media use during the pandemic, parenting stress, maternal education, satisfaction with the division of childcare, and the use of daycare will be considered as control variables.

## Materials and Methods

### Sample

This study focuses on Canadian preschool-aged children and their parents followed longitudinally at two-time points for an investigation of child digital media use during COVID-19 pandemic. Participants were recruited by distributing eye catching posters and flyers to preschools and pre-kindergarten classes, through sign-up sheets and presentations given at preschool and pre-kindergarten registration nights, a Facebook page, and newspaper and radio advertisements broadcast across Nova Scotia, Canada. At the initial assessment, the sample was composed of 316 children aged between the ages of 2 and 5 years (168 boys and 146 girls; *M* age = 3.45 years, *SD* = 0.85). This first assessment took place between April and August 2020 during a provincially declared state of emergency and lockdown. A follow-up with this sample was completed in 2021 between April and August (*N* = 266, *M* age = 4.33, *SD* = 0.86, 84% retention rate). Participants with missing data at age 4.5 did not differ from retained participants on their average screen time and effortful control scores at 3.5. Most parents were married (82%), born in Canada (91%), Caucasian (90.5%) and English-speaking (88.1%). Mothers were the primary respondents for 93.4% of the sample.

### Procedure

Parents completed the web-based Media use Questionnaire when children were 3.5 and 4.5. This assessment has been described in detail elsewhere (Barr et al., 2020). This assessment includes questions on child sex, parent education, family income, parenting stress, childcare use, and parent satisfaction with the division of childcare. For the purpose of our study, we integrated questions on child temperament using the Children’s Behavior Questionnaire – Short Form, described below. The present research was approved by Université Sainte-Anne (#0090.d) and Université de Sherbrooke’s IRB (2021–2927). Informed consent to participate was obtained from parents.

### Measures

#### Child Screen Time

Parents indicated the average amount of time children spent doing each of the following activities on weekdays and weekend days separately: (1) watching TV or DVDs; (2) using a computer; (3) playing video games on a console; (4); Using an iPad, tablet, LeapPad, iTouch, or similar mobile device (excluding smartphones); or (5) Using a smartphone. Response options included: (1) Never; (2) Less than 30 min; (3) 30 min to 1 h; (4) 1–2 h; (5) 2–3 h; (6) 4–5 h; and (7) more than 5 h. Each categorical answer was then converted to a numerical score variable reflecting the number of hours spent with each type of media. Our approach involved using the midpoint for each response range, with the exception of “5 or more hours a day” where a more conservative score of 5 was used. Weighted daily estimates were then estimated by multiplying weekday estimates by 5 and weekend day estimates by 2 and dividing the total by 7. Finally, we calculated an overall daily screen time estimate by summing average daily usage across media devices. The same procedure was used to estimate screen time at ages 3.5 and 4.5.

#### Effortful Control

Temperament was measured using the Children’s Behavior Questionnaire – Short Form (Putnam and Rothbart, 2006). This instrument measures several distinct dimensions of temperament that can be grouped into three factors: negative affectivity, surgency/extraversion, and effortful control. Effortful control was based on combined scores on the dimensions of attentional focusing (six items, i.e., Sometimes becomes absorbed in a picture book and looks at it for a long time) and inhibitory control (six items, i.e., Can wait before entering into new activities if s/he is asked to). The short version uses a 7-point Likert scale ranging from 1 (*extremely untrue of your child*) to 7 (*extremely true of your child*). Cronbach’s alphas were 0.79 and 0.79 at age 3.5 and 4.5, respectively.

#### Family Distress

Parents reported level of education, satisfaction with the division of childcare, use of childcare, and parenting stress. Education reflects the highest school grade completed by the parent. Responses were dichotomized as: (0) High school or college vocational or (1) Undergraduate or Graduate degree. Satisfaction with childcare was assessed with the following question: How satisfied are you with the division of childcare between you and your partner? Responses were recorded on a Likert scale ranging from: (1) Very satisfied; (2) Satisfied; (3) Not satisfied or unsatisfied; (4) Unsatisfied; and (5) Very unsatisfied. Parents completed the parenting distress subscale of the Parent Stress Index (Abidin, 2012). In total, parents completed 12 items (i.e., I find myself giving up more of my life to meet my child’s needs than I ever expected). Items were rated on a 5-point Likert scale as: 1 (strongly disagree); 2 (disagree); 3 (not sure); 4 (agree); or 5 (strongly agree), and were then summed to create a total score, Cronbach’s alpha = 0.85. Finally, parents reported whether or not their child was enrolled in daycare. Daycare closures were directly inferred based on the dates that daycares were ordered to close and eventually allowed to reopen^1^. Children were then categorized into three groups: (1) Daycare Non-user; (2) User daycare open; and (3) User daycare closed.

### Data Analytic Strategy

Given that greater levels of family distress are likely to contribute to greater screen time, we first considered associations between indicators of family distress and child screen media use at age 3.5 and 4.5, respectively. We then retained significant predictors of child screen media habits. To simultaneously measure associations between screen time and effortful control between the ages of 3.5 and 4.5, we then estimate a cross-lagged panel model using Mplus (Muthen and Muthen, 2018). Kline (2015) recommends achieving a ratio of *N* = 20/estimated parameter to ensure sufficient statistical power for detecting small to moderate effects in cross-lagged panel models. With a total of 15 parameter, and a sample size of 315, our study is sufficiently powered for detecting the hypothesized associations (*N* = 315 > 20 × 15 parameters).

## Results

### Descriptive Statistics

Descriptive statistics are presented in Table 1. Frequencies for categorical variables are presented in Table 2. Children spent on average *M* = 3.46 (*SD* = 2.44) and *M* = 3.25 (*SD* = 2.38) hours daily with screens at the ages of 3.5 and 4.5, respectively. As expected, child effortful control scores increased significantly between the ages of 3.5 and 4.5 [*M* = 4.71 vs 4.88) *t* (263) = 4.31, *p* < 0.001]. In total, 26% of parents had attained a high school or vocational degree. Finally, 22.3% (*n* = 59) of our sample reported not using daycare, 18.1% (*n* = 48) reported that their daycare was closed at the first assessment, and 59.6% (*n* = 158) reported that their daycare was open at the time of the first assessment.

**TABLE 1:** Descriptive statistics for continuous study measures.

Variables	*M* (*SD*)	*N*
**Age 3.5**		
Effortful control	4.70 (0.85)	315
Screen time (hours/day)	3.42 (2.44)	315
Parenting stress	27.14 (7.88)	315
Division of childcare	2.15 (1.04)	305
**Age 4.5**		
Effortful control	4.88 (0.82)	264
Screen time (hours/day)	3.25 (2.38)	265

**TABLE 2:** Frequencies for categorical variables.

Variables	%	*N*
Child sex		296
Girls	46	
Maternal education		316
High school or vocational	26	
Daycare		265
Non-user	22	
Closed	18	
Open	60	

### Family Distress and Child Screen Media Habits

We conducted a multiple linear regression to estimate the contribution of indicators of family distress to child media habits. More specifically, we regressed screen time in hours at the ages of 3.5 and 4.5 on parental education, child sex, satisfaction with the division of childcare, parenting stress, and daycare use. Regression coefficients are reported in Table 3. Lower parental education contributed to more child screen time at the ages of 3.5 (ß = 1.37, *p* < 0.001) and 4.5 (ß = 1.76, *p* < 0.001), respectively. None of the other variables were significantly related to child screen time. As such, we did not retain these variables in our cross-lagged panel model.

**TABLE 3:** Associations between family characteristics and child screen time at Ages 3.5 and 4.5.

	Screen time (in hours/day)			
Independent variables	Age 3.5		Age 4.5	
	(95% CI)	ß	B (95% CI)	ß
**Child sex**				
Girls	0.27 (−0.33–0.86)	0.05	−0.21 (−0.84–0.42)	−0.04
Boys (ref)	–		–	
**Parent education**				
High school or vocational	1.37 (0.64–2.10)*	0.23*	1.76 (0.96–2.56)*	0.30*
University degree (ref)	–			
Parenting stress	0.01 (−0.03–0.05)	0.02	0.00 (−0.04–0.04)	0.01
Division of childcare	−0.42 (−1.09–0.26)	−0.08		0.00
**Daycares**				
Non-user	−0.30 (−1.04–0.45)	−0.05	−0.44 (−1.21–0.33)	−0.08
Closed	0.28 (−0.51–1.07)	0.05	−0.28 (−1.12–0.57)	−0.04
Open (ref)	–			
R-Square	0.04		0.06	

Ref, reference group. *p ≤ 0.05.

### Cross-Lagged Panel Model

**FIGURE 1:**
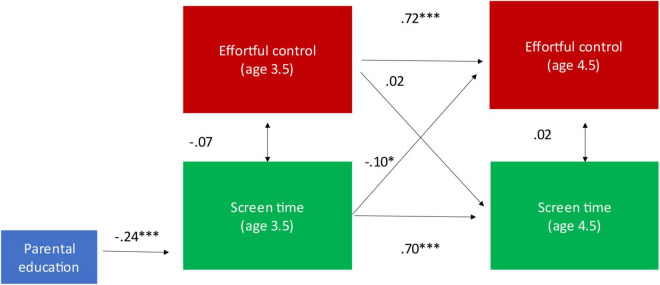
Longitudinal cross-lagged associations between preschool screen media use and effortful control. *p ≤ 0.05 and ***p ≤ 0.001.

Our model is presented in Figure 1. Our cross-lagged panel model provided good fit (CFI = 0.988; TLI = 0.965; RMSEA = 0.069 [0.000; 0.113]) and accounted for 49 and 54% of the variance in child screen time and effortful control at age 4.5, respectively. Analyses revealed considerable stability in child screen time (ß = 0.70, SE = 0.031; *p* < 0.001) and effortful control (ß = 0.72, SE = 0.030; *p* < 0.001) between the ages of 3.5 and 4.5. In terms of the cross-lagged associations, child screen time at age 3.5 significantly contributed to decreased effortful control scores at age 4.5 (ß = −0.10, SE = 0.042; *p* = 0.023) whereas effortful control at age 3.5 did not contribute to child screen time at age 4.5 (ß = 0.016, SE = 0.046; *p* = 0.729). Parental education (ß = −0.24, SE = 0.053; *p* < 0.001) was also significantly negatively associated with more child screen time at age 3.5. As indicated by the strength of the standardized coefficient, the effect size for the associations between child screen time at ages 3.5 and 4.5 (ß = 0.70) and effortful control at 3.5 and 4.5 (ß = 0.72), were large. The cross-lagged association between screen time at 3.5 and effortful control at 4.5 (ß = 0.10) was small (Cohen, 1994).

### Practical Significance

Each hour of daily screen time contributed to 10% of a standard deviation decrease in effortful control scores. Despite its small size, this association is likely to be more clinically meaningful for heavy screen media exposure. That is, for children using screen media for 4 h or more per day (32% of our sample), the contribution of daily screen time would result in 40% of a standard deviation decrease in effortful control scores. Furthermore, some have argued that even small effect sizes in psychological and behavioral medicine research can be of real-world practical significance (Rutledge and Loh, 2004).

## Discussion

The purpose of our study was to examine associations between child screen time and effortful control during the COVID-19 pandemic. Our results partially confirmed our hypotheses by indicating that higher levels of screen media intake during the pandemic at age 3.5 are prospectively associated with lower levels of effortful control at age 4.5. In contrast, lower levels of child effortful control at age 3.5 did not contribute to more screen media intake at age 4.5. Furthermore, we found partial support for the family distress hypothesis in that lower parental education was associated with higher levels of screen media use by children. Though the strength of the observed association was small, our models suggest more clinically significant effects for children exposed to high levels of screen time. These findings suggest that too much screen time media in early childhood can undermine effortful control, a key building block of personality and personal success (Kochanska and Knaack, 2003; Moffitt et al., 2011).

Theory and research suggest that child media use displaces time for self-regulation building activities such as sensitive parent-child interactions or play (Diamond and Lee, 2011). Our results indicating a prospective association over time provide support for this pathway. Future longitudinal research can seek to clarify the extent to which the displacement of children’s interactions and activities may account for the observed effects. For instance, according to one study, sedentary behavior increased among preschoolers during the pandemic whereas sleep and physical activity decreased (Alonso-Martínez et al., 2021). As such, research could examine the extent to which links between screen time and self-regulation may be accounted for by the displacement of social interactions, play, sleep, and physical activity.

The impact of digital media on young children’s development may also be driven by features of media content. The overstimulation hypothesis predicts that child media use is likely to undermine children’s development of sustained attention and ability to inhibit distractors over time (Christakis et al., 2018). In particular, this is believed to be the case because media directed at young children features a high frequency of perceptually salient elements such as frequent camera cuts and quick pacing, which are effective in eliciting child engagement without cognitive effort (Goodrich et al., 2009). Experimental research has also found that preschooler’s exposure to media content that is fantastical can deplete executive functions minutes after exposure (Lillard et al., 2015). This type of content is believed to mentally overwhelm young minds because it contradicts children’s basic understanding of the world (Smith, 2020). This would be the case, for instance, when a program or movie depicts a human character that can fly. As such, frequent exposure to fast paced and unrealistic or fantastical elements may be especially harmful.

Finally, research could examine the extent to which media use contexts including joint media engagement with parents and timing of use may further contribute to and moderate children’s development of effortful control. For instance, according to an experimental study, preschoolers assigned to view 10 episodes of the show *Daniel Tiger’s Neighborhood*, showed more gains in their empathy and emotional regulation if their parents engaged them in conversations about the content (Rasmussen et al., 2016). Furthermore, other research suggests that using media before bedtime contributes to decreased sleep quality, which then contributes to reductions in effortful control (Nathanson and Beyens, 2018). However, these associations have yet to be explored longitudinally.

By the time children enter school, the foundations of their temperaments and media habits have been established (Rothbart, 2011; Jones et al., 2013). Furthermore, early childhood interventions aimed at helping children develop healthy media habits are more likely to be effective than those undertaken with older school-aged children and adolescents (Wahi et al., 2011). For this reason, it remains important to sensitize parents and early childhood professionals that screen media can pose risks to the development of effortful control. In the context of increasing media use during the pandemic, supporting parents especially parents facing higher levels of socioeconomic vulnerability in their efforts to regulate young children’s digital media habits is especially timely. Our results reaffirm the importance of encouraging parents to establish a family media plan and to provide children with ample opportunities to engage in play and literacy building activities which can strengthen effortful control. In particular, plans can help parents implement digital media use limits for children, as well as make previsions for which contents to favor and how to accompany children’s use (Reid Chassiakos et al., 2016).

Child effortful control is narrowly linked to executive functions (Bridgett et al., 2013), school readiness (Gobeil-Bourdeau et al., 2021; Potmesilova and Potmesil, 2021), and academic competence (Liew, 2012). As such, early childhood professionals can also help support school readiness by implementing evidence-based intervention programs or strategies that benefit children’ development of skills such as inhibitory and attentional control. Furthermore, school-based programs such as *INSIGHTS into Children’s Temperament* (McClowry et al., 2005), which aim to sensitize children about their own temperaments and their challenges, have been found to be effective in reducing child behavior problems (O’Connor et al., 2014). Finally, screen time is common in childcare settings and likely to take time away from more developmentally enriching formal and informal learning opportunities (Christakis and Garrison, 2009). As such, we encourage early childhood professionals to limit their use of screen media in this setting.

### Strengths and Limitations

Strengths of the present study include our ability to establish the direction of the association between screen media use and effortful control. Furthermore, our analytical strategy also allowed us to consider developmental continuity and stability in these variables. Finally, to our knowledge, there remains limited research on the association between young children’s media habits during the COVID-19 pandemic and the development of self-regulation.

The present results are not without limitations. First, our correlational approach does not allow us to conclude that a causal association exists between child screen time and the development of effortful control. Even though we were able to provide evidence that changes in screen time habits are associated with changes in child effortful control and that changes in screen time precede changes in temperament, our design does not allow us to rule out third variable confounding. Second, as previously mentioned, we did not account for the content to which children were exposed nor did we account for the context (i.e., timing of use, adult accompaniment) in which media was used. Considering these features of media use in addition to screen time will be useful in better understanding multifinality in child outcomes. Third, both our measures of screen time and effortful control were parent reported which can introduce shared measurement bias. Fourth, our study only included two of the four possible subscales designed to measure effortful control. The inclusion of 2-year-olds in our study could also represent a limit as our temperament scale was designed for 3–7-year old’s. Nevertheless, the observed Cronbach’s alphas indicate good internal consistency. Another limit of our study is the use of a relatively homogenous, low risk convenience sample. As such, our findings may not be generalizable to the population of Canadian preschoolers. Despite this limitation, we detected a significant association between parent education and child screen time. Nonetheless, our inability to detect associations between child effortful control and later screen time habits could reflect the fact that our sample is relatively homogenous in terms of its demographic characteristics. Previous studies have found that child temperamental characteristics such as surgency and negative affectivity predict preschooler screen time in contexts of high social risks, defined by low maternal education, income, and higher levels of maternal depression (McArthur et al., 2022). Future research should seek to examine these associations in larger more diverse samples. Last, although our sample was sufficiently powered for our analyses, we were limited in our ability to detect small effects.

## Conclusion

To our knowledge, this is the first study to examine how higher amounts of preschooler screen time are prospectively associated with decreases in effortful control in the context of a pandemic. Our results suggest that screen media use during early childhood, a sensitive period for the development of lifelong temperament, should be closely monitored by parents. Furthermore, our findings add to the literature suggesting that limiting screen time during the preschool period may benefit child socio-emotional and school readiness skills.

## Data Availability Statement

The data presented in this article are not readily available. As per the participant consent form, data are only available to the research team. Requests to access the data should be directed to caroline.fitzpatrick@usherbrooke.ca.

## Ethics Statement

The studies involving human participants were reviewed and approved by Comité d’Étique, Université Sainte-Anne; Comité d’Étique, Université de Sherbrooke. Written informed consent to participate in this study was provided by the participants’ legal guardian/next of kin.

## Author Contributions

CF designed the study and drafted most of the manuscript. GG-C conducted the analyses and EC drafted the methods. EH, AL, J-PL provided critical theoretical feedback on the entire manuscript. All authors have read and approved the manuscript.

## Conflict of Interest

The authors declare that the research was conducted in the absence of any commercial or financial relationships that could be construed as a potential conflict of interest.

## Publisher’s Note

All claims expressed in this article are solely those of the authors and do not necessarily represent those of their affiliated organizations, or those of the publisher, the editors and the reviewers. Any product that may be evaluated in this article, or claim that may be made by its manufacturer, is not guaranteed or endorsed by the publisher.

## Footnotes

1
https://novascotia.ca/news/release/?id=20200313009

## References

[B1] Abidin R. R. (2012). *Parenting Stress Index*, 4th Edn. Lutz, FL: PAR.

[B2] Alonso-Martínez A. M. Ramírez-Vélez R. García-Alonso Y. Izquierdo M. García-Hermoso A. (2021). Physical activity, sedentary behavior, sleep and self-regulation in Spanish preschoolers during the COVID-19 lockdown. *Int. J. Environ. Res. Public Health* 18:693. 10.3390/ijerph18020693 33467399 PMC7830291

[B3] Asplund K. M. Kair L. R. Arain Y. H. Cervantes M. Oreskovic N. M. Zuckerman K. E. (2015). Early childhood screen time and parental attitudes toward child television viewing in a low-income Latino population attending the special supplemental nutrition program for women, infants, and children. *Childh. Obes.* 11 590–599. 10.1089/chi.2015.0001 26390321 PMC4628228

[B4] Barr R. Kirkorian H. Radesky J. Coyne S. Nichols D. Blanchfield O. (2020). Beyond screen time: a synergistic approach to a more comprehensive assessment of family media exposure during early childhood. *Front. Psychol.* 11:1283. 10.3389/fpsyg.2020.01283 32754078 PMC7365934

[B5] Blair C. Raver C. C. (2015). School readiness and self-regulation: a developmental psychobiological approach. *Annu. Rev. Psychol.* 66 711–731. 10.1146/annurev-psych-010814-015221 25148852 PMC4682347

[B6] Blair C. Razza R. P. (2007). Relating effortful control, executive function, and false belief understanding to emerging math and literacy ability in kindergarten. *Child Dev.* 78 647–663. 10.1111/j.1467-8624.2007.01019.x 17381795

[B7] Bridgett D. J. Oddi K. B. Laake L. M. Murdock K. W. Bachmann M. N. (2013). Integrating and differentiating aspects of self-regulation: effortful control, executive functioning, and links to negative affectivity. *Emotion* 13 47–63. 10.1037/a0029536 22906086

[B8] Christakis D. A. Garrison M. M. (2009). Preschool-aged children’s television viewing in child care settings. *Pediatrics* 124 1627-1632. 10.1542/peds.2009-0862 19933733

[B9] Christakis D. A. Ramirez J. S. B. Ferguson S. M. Ravinder S. Ramirez J. M. (2018). How early media exposure may affect cognitive function: a review of results from observations in humans and experiments in mice. *Proc. Natl. Acad. Sci. U.S.A.* 115 9851-9858. 10.1073/pnas.1711548115 30275319 PMC6176595

[B10] Clifford S. Doane L. D. Breitenstein R. Grimm K. J. Lemery-Chalfant K. (2020). Effortful control moderates the relation between electronic-media use and objective sleep indicators in childhood. *Psychol. Sci.* 31 822–834. 10.1177/0956797620919432 32558622 PMC7492726

[B11] Cohen J. (1994). The earth is round (*p* < .05). *Am. Psychol.* 49 997–1003.

[B12] Coyne S. M. Shawcroft J. Gale M. Gentile D. A. Etherington J. T. Holmgren H. (2021). Tantrums, toddlers and technology: temperament, media emotion regulation, and problematic media use in early childhood. *Comput. Human Behav.* 120:106762. 10.1016/j.chb.2021.106762 33927469 PMC8078852

[B13] Diamond A. (2002). “Normal development of prefrontal cortex from birth to young adulthood: cognitive functions, anatomy, and biochemistry,” in *Principles of Frontal Lobe Function*, eds Stuss D. Knight R. (New York, NY: Oxford), 466–503.

[B14] Diamond A. Lee K. (2011). Interventions shown to aid executive function development in children 4 to 12 years old. *Science* 333 959-964. 10.1126/science.1204529 21852486 PMC3159917

[B15] Diamond A. Barnett W. S. Thomas J. Munro S. (2007). Preschool program improves cognitive control. *Science* 318 1387–1388. 10.1126/science.1151148 18048670 PMC2174918

[B16] Diaz A. Eisenberg N. Valiente C. VanSchyndel S. Spinrad T. L. Berger R. (2017). Relations of positive and negative expressivity and effortful control to kindergarteners’ student–teacher relationship, academic engagement, and externalizing problems at school. *J. Res. Pers.* 67 3–14. 10.1016/j.jrp.2015.11.002 28584388 PMC5455333

[B17] Eisenberg N. Taylor Z. E. Widaman K. F. Spinrad T. L. (2015). Externalizing symptoms, effortful control, and intrusive parenting: a test of bidirectional longitudinal relations during early childhood. *Dev. Psychopathol.* 27(4pt1) 953–968. 10.1017/S0954579415000620 26439056

[B18] Eisenberg N. Valiente C. Spinrad T. L. Cumberland A. Liew J. Reiser M. (2009). Longitudinal relations of children’s effortful control, impulsivity, and negative emotionality to their externalizing, internalizing, and co-occurring behavior problems. *Dev. Psychol.* 45 988–1008. 10.1037/a0016213 19586175 PMC2775424

[B19] Fitzpatrick C. Almeida M. L. Harvey E. Garon-Carrier G. Berrigan F. Asbridge M. (2022). An examination of bedtime media and excessive screen time by Canadian preschoolers during the COVID-19 pandemic. *BMC Pediatr.* 22:212. 10.1186/s12887-022-03280-8 35436899 PMC9418412

[B20] Gartstein M. A. Putnam S. P. Rothbart M. K. (2012). Etiology of preschool behavior problems: contributions of temperament attributes in early childhood. *Infant Ment. Health J.* 33 197–211. 10.1002/imhj.21312 28520102

[B21] Gentile D. A. Choo H. Liau A. Sim T. Li D. Fung D. (2011). Pathological video game use among youths: a two-year longitudinal study. *Pediatrics* 127 e319–e329. 10.1542/peds.2010-1353 21242221

[B22] Gobeil-Bourdeau J. Lemelin J.-P. Letarte M.-J. Laurent A. (2021). Can temperament predict school readiness in at-risk kindergarteners? A combination of variable-oriented and person-oriented approaches. *Early Educ. Dev.* 1–20. 10.1080/10409289.2021.194763335082478

[B23] Goodrich S. A. Pempek T. A. Calvert S. L. (2009). Formal production features of infant and toddler DVDs. *Arch. Pediatr. Adolesc. Med.* 163 1151–1156. 10.1001/archpediatrics.2009.201 19996053

[B24] Hartshorne J. K. Huang Y. T. Paredes P. M. L. Oppenheimer K. Robbins P. T. Velasco M. D. (2021). Screen time as an index of family distress. *Curr. Res. Behav. Sci.* 2:100023. 10.1016/j.crbeha.2021.100023

[B25] Hutton J. S. Dudley J. Horowitz-Kraus T. DeWitt T. Holland S. K. (2020). Associations between screen-based media use and brain white matter integrity in preschool-aged children. *JAMA Pediatr.* 174:e193869. 10.1001/jamapediatrics.2019.3869 31682712 PMC6830442

[B26] Jonas K. Kochanska G. (2018). An imbalance of approach and effortful control predicts externalizing problems: support for extending the dual-systems model into early childhood. *J. Abnorm. Child Psychol.* 46 1573–1583. 10.1007/s10802-018-0400-3 29372367 PMC6060018

[B27] Jones R. A. Hinkley T. Okely A. D. Salmon J. (2013). Tracking physical activity and sedentary behavior in childhood: a systematic review. *Am. J. Prevent. Med.* 44 651–658. 10.1016/j.amepre.2013.03.001 23683983

[B28] Kline R. B. (2015). *Principles and Practice of Structural Equation Modeling.* New York, NY: Guilford publications.

[B29] Kochanska G. Knaack A. (2003). Effortful control as a personality characteristic of young children: antecedents, correlates, and consequences. *J. Pers.* 71 1087–1112. 10.1111/1467-6494.7106008 14633059

[B30] Konok V. Liszkai-Peres K. Bunford N. Ferdinandy B. Jurányi Z. Ujfalussy D. J. (2021). Mobile use induces local attentional precedence and is associated with limited socio-cognitive skills in preschoolers. *Comput. Hum. Behav.* 120:106758. 10.1016/j.chb.2021.106758

[B31] Landry S. H. Zucker T. A. Williams J. M. Merz E. C. Guttentag C. L. Taylor H. B. (2017). Improving school readiness of high-risk preschoolers: combining high quality instructional strategies with responsive training for teachers and parents. *Early Childh. Res. Q.* 40 38–51. 10.1016/j.ecresq.2016.12.001

[B32] Lengua L. J. (2012). “Poverty, the development of effortful control, and children’s academic, social, and emotional adjustment,” in *The Oxford Handbook of Poverty and Child Development*, eds Maholmes V. King R. B. (Oxford: Oxford University Press), 491–511.

[B33] Liew J. (2012). Effortful control, executive functions, and education: Bringing self-regulatory and social-emotional competencies to the table. *Child Dev. Perspect.* 6 105–111. 10.1111/j.1750-8606.2011.00196.x

[B34] Lillard A. S. Drell M. B. Richey E. M. Boguszewski K. Smith E. D. (2015). Further examination of the immediate impact of television on children’s executive function. *Dev. Psychol.* 51 792–805. 10.1037/a0039097 25822897

[B35] Madigan S. Browne D. Racine N. Mori C. Tough S. (2019). Association between screen time and children’s performance on a developmental screening test. *JAMA Pediatr.* 173 244–250. 10.1001/jamapediatrics.2018.5056 30688984 PMC6439882

[B36] Madigan S. Racine N. Tough S. (2020). Prevalence of preschoolers meeting vs exceeding screen time guidelines. *JAMA Pediatr.* 174 93–95. 10.1001/jamapediatrics.2019.4495 31764965 PMC6902205

[B37] McArthur B. A. Hentges R. Christakis D. A. McDonald S. Tough S. Madigan S. (2022). Cumulative social risk and child screen use: the role of child temperament. *J. Pediatr. Psychol.* 47 171–179. 10.1093/jpepsy/jsab087 34388254 PMC8841983

[B38] McClowry S. Snow D. L. Tamis-LeMonda C. S. (2005). An evaluation of the effects of INSIGHTS on the behavior of inner city primary school children. *J. Prim. Prevent.* 26 567–584. 10.1007/s10935-005-0015-7 16237502 PMC1425905

[B39] Merz E. C. Landry S. H. Williams J. M. Barnes M. A. Eisenberg N. Spinrad T. L. (2014). Associations among parental education, home environment quality, effortful control, and preacademic knowledge. *J. Appl. Dev. Psychol.* 35 304–315. 10.1016/j.appdev.2014.04.002 25110382 PMC4121596

[B40] Moffitt T. E. (1993). Adolescence-limited and life-course-persistent antisocial behavior: a developmental taxonomy. *Psychol. Rev.* 100 674–701. 10.1037/0033-295X.100.4.674 8255953

[B41] Moffitt T. E. Arseneault L. Belsky D. Dickson N. Hancox R. J. Harrington H. (2011). A gradient of childhood self-control predicts health, wealth, and public safety. *Proc. Natl. Acad. Sci. U.S.A.* 108 2693–2698. 10.1073/pnas.1010076108 21262822 PMC3041102

[B42] Montroy J. J. Bowles R. P. Skibbe L. E. McClelland M. M. Morrison F. J. (2016). The development of self-regulation across early childhood. *Dev. Psychol.* 52 1744–1762. 10.1037/dev0000159 27709999 PMC5123795

[B43] Morris A. S. John A. Halliburton A. L. Morris M. D. Robinson L. R. Myers S. S. (2013). Effortful control, behavior problems, and peer relations: what predicts academic adjustment in kindergartners from low-income families? *Early Educ. Dev.* 24 813–828. 10.1080/10409289.2013.744682 24163572 PMC3806504

[B44] Murray K. T. Kochanska G. (2002). Effortful control: Factor structure and relation to externalizing and internalizing behaviors. *J. Abnorm. Child Psychol.* 30 503–514. 10.1023/A:1019821031523 12403153

[B45] Muthen L. K. Muthen B. O. (2018). *Mplus User’s Guide*, 7th Edn. Los Angeles, CA: Muthen & Muthen.

[B46] Nathanson A. I. Aladé F. Sharp M. L. Rasmussen E. E. Christy K. (2014). The relation between television exposure and executive function among preschoolers. *Dev. Psychol.* 50 1497–1506. 10.1037/a0035714 24447117

[B47] Nathanson A. I. Beyens I. (2018). The role of sleep in the relation between young children’s mobile media use and effortful control. *Br. J. Dev. Psychol.* 36 1–21. 10.1111/bjdp.12196 28792067

[B48] Nix R. L. Bierman K. L. Motamedi M. Heinrichs B. S. Gill S. (2018). Parent engagement in a Head Start home visiting program predicts sustained growth in children’s school readiness. *Early Childh. Res. Q.* 45 106–114. 10.1016/j.ecresq.2018.06.006 30911204 PMC6430128

[B49] O’Connor E. E. Cappella E. McCormick M. P. McClowry S. G. (2014). An examination of the efficacy of INSIGHTS in enhancing the academic and behavioral development of children in early grades. *J. Educa. Psychol.* 106 1156–1169. 10.1037/a0036615

[B50] Olson S. L. Sameroff A. J. Kerr D. C. Lopez N. L. Wellman H. M. (2005). Developmental foundations of externalizing problems in young children: the role of effortful control. *Dev. Psychopathol.* 17 25–45. 10.1017/S0954579405050029 15971758

[B51] Pagani L. S. Fitzpatrick C. Barnett T. A. (2013). Early childhood television viewing and kindergarten entry readiness. *Pediatr. Res.* 74 350–355. 10.1038/pr.2013.105 23788060

[B52] Park Y. R. Nix R. L. Gill S. Hostetler M. L. (2022). What kind of parenting is associated with early self-control among toddlers living in poverty? The importance of learning support. *Dev. Psychol.* 58 425–437. 10.1037/dev0001312 35007108 PMC10103748

[B53] Parrish K. H. Smith M. R. Moran L. Ruberry E. J. Lengua L. J. (2022). Tests of bidirectional relations of TV exposure and effortful control as predictors of adjustment in early childhood in the context of family risk factors. *Infant Child Dev.* e2314. 10.1002/icd.2314 PMC9432819 36060792

[B54] Paulus F. W. Sinzig J. Mayer H. Weber M. von Gontard A. (2018). Computer gaming disorder and ADHD in young children—a population-based study. *Int. J. Ment. Health Addict.* 16 1193–1207. 10.1007/s11469-017-9841-0

[B55] Potmesilova P. Potmesil M. (2021). Temperament and school readiness – a literature review. *Front. Psychol.* 12:599411. 10.3389/fpsyg.2021.599411 34093300 PMC8172806

[B56] Putnam S. P. Rothbart M. K. (2006). Development of short and very short forms of the children’s behavior questionnaire. *J. Pers. Assess.* 87 102–112. 10.1207/s15327752jpa8701_09 16856791

[B57] Rasmussen E. E. Shafer A. Colwell M. J. White S. Punyanunt-Carter N. Densley R. L. (2016). Relation between active mediation, exposure to Daniel Tiger’s Neighborhood, and US preschoolers’ social and emotional development. *J. Child. Media* 10 443–461. 10.1080/17482798.2016.1203806

[B58] Reid Chassiakos Y. L. Radesky J. Christakis D. Moreno M. A. Cross C. Hill D. (2016). Children and adolescents and digital media. *Pediatrics* 138:e20162593. 10.1542/peds.2016-2593 27940795

[B59] Ribner A. Fitzpatrick C. Blair C. (2017). Family socioeconomic status moderates associations between television viewing and school readiness skills. *J. Dev. Behav. Pediatr.* 38 233–239. 10.1097/DBP.0000000000000425 28240651

[B60] Rideout V. (2020). *The Common Sense Census: Media use by Kids Age Zero to Eight.* San Francisco, CA: Common Sense Media.

[B61] Rothbart M. K. (2011). *Becoming Who We Are: Temperament and Personality in Development.* New York, NY: Guilford Press.

[B62] Rothbart M. K. Bates J. E. (2006). “Temperament,” in *Handbook of Child Psychology: Social, Emotional, and Personality Development*, 6th Edn, Vol. 3 eds Eisenberg N. Damon W. Lerner R. (Hoboken, NJ: John Wiley & Sons Inc.), 99–166.

[B63] Rutledge T. Loh C. (2004). Effect sizes and statistical testing in the determination of clinical significance in behavioral medicine research. *Ann. Behav. Med.* 27 138–145. 10.1207/s15324796abm2702_9 15026298

[B64] Sánchez-Pérez N. Fuentes L. J. Eisenberg N. González-Salinas C. (2018). Effortful control is associated with children’s school functioning via learning-related behaviors. *Learn. Individ. Differ.* 63 78–88. 10.1016/j.lindif.2018.02.009

[B65] Santens E. Claes L. Dierckx E. Dom G. (2020). Effortful control–A transdiagnostic dimension underlying internalizing and externalizing psychopathology. *Neuropsychobiology* 79 255–269. 10.1159/000506134 32106115

[B66] Shin E. Choi K. Resor J. Smith C. L. (2021). Why do parents use screen media with toddlers? The role of child temperament and parenting stress in early screen use. *Infant Behav. Dev.* 64:101595. 10.1016/j.infbeh.2021.101595 34153781

[B67] Smith C. L. Day K. L. (2018). Parenting, anger, and effortful control as predictors of child externalizing behavior: the role of child sex as a moderator. *Int. J. Behav. Dev.* 42 248–256. 10.1177/0165025417692898

[B68] Smith H. (2020). *Children, Executive Functioning, and Digital Media a Review.* San Francisco, CA: Common Sense Media.

[B69] Tamana S. K. Ezeugwu V. Chikuma J. Lefebvre D. L. Azad M. B. Moraes T. J. (2019). Screen-time is associated with inattention problems in preschoolers: results from the CHILD birth cohort study. *PLoS One* 14:e0213995. 10.1371/journal.pone.0213995 30995220 PMC6469768

[B70] Thompson A. L. Adair L. S. Bentley M. E. (2013). Maternal characteristics and perception of temperament associated with infant TV exposure. *Pediatrics* 131 e390–e397. 10.1542/peds.2012-1224 23296440 PMC3557404

[B71] Tiberio S. S. Capaldi D. M. Kerr D. C. R. Bertrand M. Pears K. C. Owen L. (2016). Parenting and the development of effortful control from early childhood to early adolescence: a transactional developmental model. *Dev. Psychopathol.* 28 837-853. 10.1017/S0954579416000341 27427809 PMC5206969

[B72] Valiente C. Lemery-Chalfant K. Swanson J. Reiser M. (2008). Prediction of children’s academic competence from their effortful control, relationships, and classroom participation. *J. Educ. Psychol.* 100 67–77. 10.1037/0022-0663.100.1.67 21212831 PMC3014585

[B73] Wahi G. Parkin P. C. Beyene J. Uleryk E. M. Birken C. S. (2011). Effectiveness of interventions aimed at reducing screen time in children: a systematic review and meta-analysis of randomized controlled trials. *Arch. Pediatr. Adolesc. Med.* 165 979–986. 10.1001/archpediatrics.2011.122 21727260

[B74] Walton K. Simpson J. R. Darlington G. Haines J. (2014). Parenting stress: a cross-sectional analysis of associations with childhood obesity, physical activity, and TV viewing. *BMC Pediatr.* 14:244. 10.1186/1471-2431-14-244 25270356 PMC4194416

[B75] Wang F. L. Eisenberg N. Valiente C. Spinrad T. L. (2016). Role of temperament in early adolescent pure and co-occurring internalizing and externalizing problems using a bifactor model: moderation by parenting and gender. *Dev. Psychopathol.* 28(4pt2) 1487–1504. 10.1017/S0954579415001224 26646352 PMC4900935

[B76] Warren S. M. Barnett M. A. (2020). Effortful control development in the face of harshness and unpredictability. *Hum. Nat.* 31 68–87. 10.1007/s12110-019-09360-6 31898018

[B77] Wichstrøm L. Penelo E. Rensvik Viddal K. de la Osa N. Ezpeleta L. (2018). Explaining the relationship between temperament and symptoms of psychiatric disorders from preschool to middle childhood: Hybrid fixed and random effects models of Norwegian and Spanish children. *J. Child Psychol. Psychiatry* 59 285–295. 10.1111/jcpp.12772 28671298

[B78] Wright J. C. Huston A. C. Vandewater E. A. Bickham D. S. Scantlin R. M. Kotler J. A. (2001). American children’s use of electronic media in 1997: a national survey. *J. Appl. Dev. Psychol.* 22 31–47.

[B79] Zalewski M. Lengua L. J. Fisher P. A. Trancik A. Bush N. R. Meltzoff A. N. (2012). Poverty and single parenting: relations with preschoolers’ cortisol and effortful control. *Infant Child Dev.* 21 537–554. 10.1002/icd.1759

[B80] Zhou Q. Chen S. H. Main A. (2012). Commonalities and differences in the research on Children’s effortful control and executive function: a call for an integrated model of self- regulation. *Child Dev. Perspect.* 6 112–121. 10.1111/j.1750-8606.2011.00176.x

